# Co-linearity and divergence of the A subgenome of *Brassica juncea* compared with other *Brassica* species carrying different A subgenomes

**DOI:** 10.1186/s12864-015-2343-1

**Published:** 2016-01-05

**Authors:** Jun Zou, Dandan Hu, Peifa Liu, Harsh Raman, Zhongsong Liu, Xianjun Liu, Isobel A. P. Parkin, Boulos Chalhoub, Jinling Meng

**Affiliations:** National Key Laboratory of Crop Genetic Improvement, Key Laboratory of Rapeseed Genetic Improvement, Ministry of Agriculture P. R. China, Huazhong Agricultural University, Wuhan, 430070 China; Graham Centre for Agricultural Innovation (an alliance between the Charles Sturt University and NSW Department of Primary Industries), Wagga Wagga Agricultural Institute, Wagga Wagga, NSW 2650 Australia; Oilseed Crops Institute, Hunan Agricultural University, Changsha, 410128 China; Agriculture and Agri-Food Canada, 107 Science Place, Saskatoon, SK S7N 0X2 Canada; Unité de Recherche en Génomique Végétale (Institut National de la Recherche Agronomique, Centre National de la Recherche Scientifique, Université d’Evry Val d’Essonnes), Organization and Evolution of Plant Genomes, 91057 Evry cedex, France

**Keywords:** *Brassica juncea*, Dense genetic map, Subgenome, Genome organization, Co-linearity, Divergence

## Abstract

**Background:**

There are three basic *Brassica* genomes (A, B, and C) and three parallel sets of subgenomes distinguished in the diploid *Brassica* (i.e.: *B. rapa*, A^r^A^r^; *B. nigra*, B^ni^B^ni^; *B. oleracea*, C^o^C^o^) and the derived allotetraploid species (i.e.: *B. juncea*, A^j^A^j^B^j^B^j^; *B. napus*, A^n^A^n^C^n^C^n^; *B. carinata,* B^c^B^c^C^c^C^c^). To understand subgenome differentiation in *B. juncea* in comparison to other A genome-carrying *Brassica* species (*B. rapa* and *B. napus*), we constructed a dense genetic linkage map of *B. juncea*, and conducted population genetic analysis on diverse lines of the three A-genome carrying *Brassica* species using a genotyping-by-sequencing approach (DArT-seq).

**Results:**

A dense genetic linkage map of *B. juncea* was constructed using an F_2_ population derived from Sichuan Yellow/Purple Mustard. The map included 3329 DArT-seq markers on 18 linkage groups and covered 1579 cM with an average density of two markers per cM. Based on this map and the alignment of the marker sequences with the physical genome of *Arabidopsis thaliana*, we observed strong co-linearity of the ancestral blocks among the different A subgenomes but also considerable block variation. Comparative analyses at the level of genome sequences of *B. rapa* and *B. napus*, and marker sequence anchored on the genetic map of *B. juncea*, revealed a total of 30 potential inversion events across large segments and 20 potential translocation events among the three A subgenomes. Population genetic analysis on 26 accessions of the three A genome-carrying *Brassica* species showed that the highest genetic distance were estimated when comparing A^j^-A^n^ than between A^n^-A^r^ and A^j^-A^r^ subgenome pairs.

**Conclusions:**

The development of the dense genetic linkage map of *B. juncea* with informative DArT-seq marker sequences and availability of the reference sequences of the A^r^, and A^n^C^n^ genomes allowed us to compare the A subgenome structure of *B. juncea* (A^j^) . Our results suggest that strong co-linearity exists among the three A *Brassica* genomes (A^r^, A^n^ and A^j^) but with apparent subgenomic variation. Population genetic analysis on three A-genome carrying *Brassica* species support the idea that *B. juncea* has distinct genomic diversity, and/or evolved from a different A genome progenitor of *B. napus*.

**Electronic supplementary material:**

The online version of this article (doi:10.1186/s12864-015-2343-1) contains supplementary material, which is available to authorized users.

## Background

High-throughput sequencing techniques have enabled researchers to construct dense genetic linkage maps for various agricultural crops [1–4]. These high-density maps not only give detailed resolution of genomes but also provide excellent platforms to identify molecular markers for qualitative and quantitative loci associated with trait(s) of interest. Genome-wide comparative analyses allow the elucidation of chromosomal rearrangements resulting from speciation, genome evolution, and adaptation as well as the identification of novel alleles for genetic improvement within and among crop species [5, 6]. A number of genetic linkage maps of species in the Brassicaceae family have been generated over the past 20 years, largely focusing on commercially important *Brassica* species. These genetic linkage maps were based on traditional DNA markers such as restriction fragment length polymorphism (RFLP), amplified fragment length polymorphism (AFLP) and simple sequence repeats (SSR) [7–13], and high-throughput RNA and DNA sequence markers detected by single nucleotide polymorphism (SNP) arrays, re-sequencing or genotyping-by-sequencing technologies [2, 4, 14–17].

Comparative mapping studies with the well-characterized relative *Arabidopsis thaliana* revealed a common hexaploid ancestor in the lineage of the Brassicas. These mapping studies also identified 21–24 conserved ancestral blocks of the Brassicaceae [5, 6, 8, 18–21]. These blocks have been used extensively in comparative genomic analyses among *Brassica* species. Genome-wide analyses between the allotetraploid *B. napus* and its diploid progenitors *B. rapa* and *B. oleracea* have shown significant genomic co-linearity [5, 6], and functional conservation of genetic loci governing important traits has also been revealed between different A subgenomes [22]; however, changes in the subgenome resulting from events such as genome duplication, inversion and homoeologous exchange have been documented in *B. napus* [23, 24].

In the U’s triangle of *Brassica* [25], there are three basic genomes (A, B and C), i.e. A^r^ in *B. rapa*, B^ni^ in *B. nigra*, C^o^ in *B. olereaca*, and three sets of corresponding subgenomes in the cultivated allotetraploid oilseed*Brassica* species, i.e. A^j^B^j^ of *B. juncea* (Indian or Oriental mustard, 2n = 4 × = 36), A^n^C^n^ of *B. napus* (rapeseed, 2n = 4 × = 38), and B^c^C^c^ of *B. carinata* (Ethiopian mustard, 2n = 4 × = 34). Genome differentiation, due to translocation, inversion, deletion, duplication, and transposon activation would be expected to have occurred among the different subgenomes in these polyploid *Brassica* species as a result of genomic shock during interspecific hybridization, and long-term domestication and cultivation [23, 26–29]. The extensive genetic diversity occurring within these agriculturally important oilseed species has been exploited to create novel germplasm resources in pre-breeding programs [30–32]. Understanding the genetic basis of the subgenomic variation among the three sets of *Brassica* subgenomes may provide insights for genomics-based rapeseed breeding programs involving favorable allele introgression from allied species.

In the present study, we focus on *B. juncea*, an ancient oilseed crop which is acknowledged for its suitability for cultivation under moisture-limited (drought) and hot conditions. *B. juncea* also harbors loci for resistance to blackleg disease, which is caused by the fungus *Leptosphaeria maculans* [33]. *B. juncea* has been grown widely for oil and protein in Asia, especially India and China, and other parts of the world for approximately 6000 years [34]. However, genetic and genomic resources for *B. juncea* are scarce compared with the resources for the major rapeseed crop, *B. napus*. The first genetic linkage map of *B. juncea* was constructed with 343 RFLP markers using a mapping population derived from two Canadian cultivars [11]. Subsequently, several genetic linkage maps have been constructed using mapping populations originating from Canada, India and Europe by SSR and AFLP markers [12, 13, 35, 36]. Recently, a high-density genetic linkage map of *B. juncea* was developed using RNA-based SNP markers [16].

The availability of the linkage and comparative maps of bi-parental populations of *B. juncea* [9, 13, 35–37], and the reference sequences of the A^r^ genome of *B. rapa*, and the A^n^ genome of *B. napus* [23, 38], has made it possible to analyze the subgenomic variation among different A genomes of *B. juncea*, *B. napus* and*B. rapa*. Here, we report the construction of a dense genetic linkage map of *B. juncea* generated using an F_2_ population, derived from Sichuan Yellow (SY) and Purple Mustard (PM) [37] designated as SY-PM population, with high-throughput markers based on the genotyping-by-sequencing approach, DArT-seq [15, 39]. We also investigated allelic diversity and population structure among 26 genotypes of three *Brassica* species carrying A genomes (i.e., *B. juncea, B. rapa* and *B. napus*) that represent different geographic origins. We compared the genome-wide arrangement of sequences and the constitution of the 24 ancestral genomic blocks (A–X) of Brassicaceae in *B. juncea*, *B. rapa* and *B. napus*. These results would provide valuable resources for *Brassica* genomic studies, especially for understanding and exploring subgenomic variation among different *Brassica* species.

## Results

### Construction of a dense genetic linkage map of *B. juncea*

We used a set of 4833 representative markers, selected from 6836 identified DArT-seq polymorphic markers with less than 10 % missing rate, to construct the genetic map for SY-PM population. A total of 3329 of the markers could be assigned into 18 large linkage groups, while the remaining 1504 markers which could not be linked to any of the large groups were discarded. The 3329 linked markers detected 1570 discrete genetic loci on 18 linkage groups and covered 1579 cM (centiMorgan) with an average density of two markers per cM. Seventy-two percent (1132) of the loci were detected by single markers, while 438 were defined by multiple markers and were therefore defined as genetic “bin loci” (Table 1, Additional files 1 and 2).

**Table 1:** Characteristics of linkage groups of an intercross SY-PM population of *B. juncea*

Linkage group (LG)	No. of single-marker loci	No. of bin loci	Total number of loci	Total number of mapped markers	Coverage (cM)
A01	62	32	94	274	91
A02	75	21	96	189	72.3
A03	72	26	98	163	94.9
A04	48	13	61	133	63.3
A05	71	24	95	150	94.4
A06	80	25	105	160	98.2
A07	40	31	71	163	71.5
A08	59	26	85	177	66.4
A09	62	38	100	311	108
A10	71	12	83	144	68.4
Subtotal for the A subgenome	640	248	888	1864	828.4
B01	41	26	67	171	62.8
B02	67	39	106	236	105.1
B03	69	17	86	230	59.8
B04	66	17	83	139	115.4
B05	51	19	70	125	102.2
B06	50	11	61	79	75.8
B07	60	39	99	246	102.2
B08	88	22	110	239	126.7
Subtotal for the B subgenome	492	190	682	1465	750
Total for the AB genome	1132	438	1570	3329	1578.5
Mean (per LG)	62.9	24.3	87.2	184.9	87.7

**Fig. 1:**
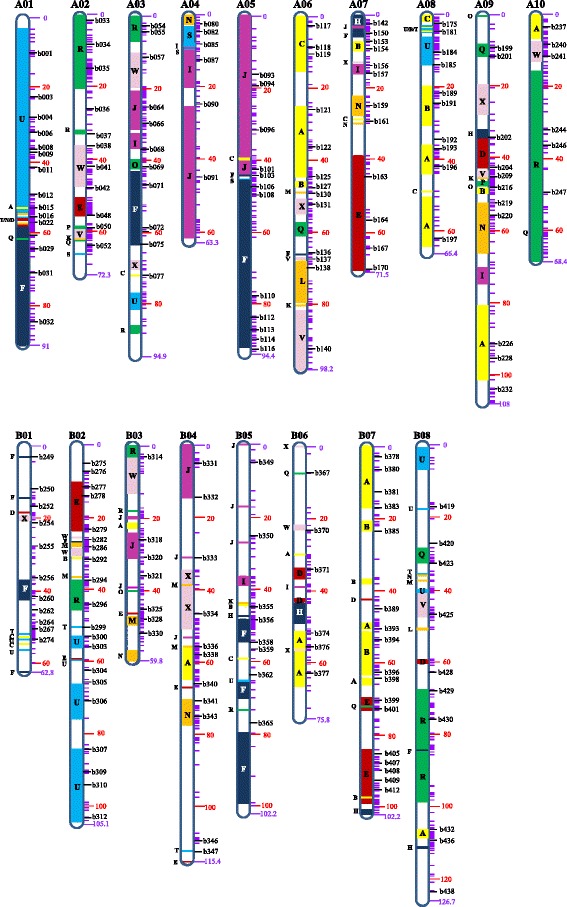
The genetic linkage map of the SY-PM population of *B. junce*a. Genetic loci are shown with purple bars (single marker loci) and longer black bars (bin loci) with code number. The genetic distance for a locus is indicated for the first and last bars on each linkage group, in addition to the red scale bars. The conserved ancestral blocks of Brassicaceae are labeled as A to X and are shown on the body of each linkage group except the small blocks (distance ≤2 cM) which are shown on the left side of each chromosome

The 3329 mapped markers were used for comparative genomic analysis. In total, 1031 marker sequences representing 787 genetic loci could be aligned with the genome sequence of *A. thaliana* (Additional file 2), which were used to discriminate the constitution of the conserved ancestral Brassicaceae blocks in the genome of *B. juncea*. A total of 78 block-units were identified in the dense genetic map of the SY-PM population (Fig. 1). Five blocks (i.e., A, F, J, R and U), each of which had an average of 6 copies, often covered large genetic regions spanning 20 cM or more (e.g., large F blocks on the A01, A03, A05, and B05 linkage groups) in the SY-PM map.

### Co-linearity analysis between the A-subgenomes of different *Brassica* species

Conserved *Brassica* blocks that were identified in the A^j^ subgenome of *B. juncea* through genetic mapping (see above) were used for co-linearity analysis with the two sequenced A genomes, i.e. A^r^ and A^n^ subgenome [23, 38]. Blocks A, B and I (four copies) were the most prevalent blocks in the A^j^ subgenome, while the blocks B (eight copies) and T (seven copies) were the two most abundant blocks in the A^r^ and A^n^ subgenomes (Additional file 3).

**Fig. 2:**
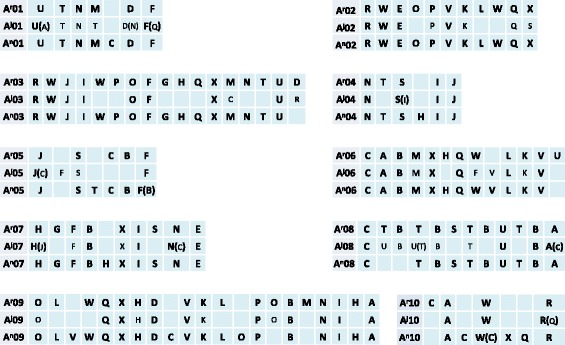
Block arrangement and ancestral karyotypes in three A subgenomes of *Brassica* species. Comparison were made among the A^r^ subgenome in *B. rapa*, A^n^ in *B. napus*, and A^j^ in *B. juncea*. The constitution of the ancestral blocks in each subgenome is arranged correspondingly. The blocks in the brackets indicate blocks or small insertion that varied among different genetic maps for the species

Strong co-linearity was found among the three A subgenomes, while a few conserved ancestral blocks were apparently subjected to inversions, deletions, and shuffling (blocks exchange) (Fig. 2). Comparing the A^r^ and A^n^ genomes, for example, there was an inversion between blocks O and P in chromosome A09 companied by deletion of block M on chromosome A^n^09, but deletion of block V in A^r^09, and a duplication of block T and B was observed in A^r^08 compared to A^n^08. The restricted alignment of the marker sequences to the ancestral blocks and the limitations of genetic mapping make it difficult to confirm the absence of blocks in SY-PM genetic map. However, several small insertions in the ancestral blocks of *B. juncea* could be observed, e.g. a small segment from block C was inserted in block J on A^j^05.

**Fig. 3:**
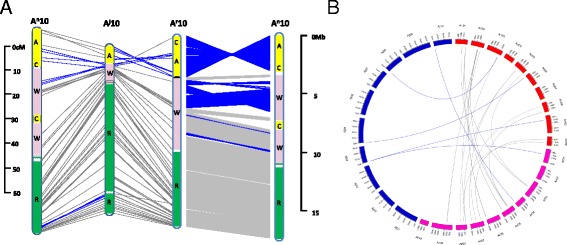
Chromosomal structural variations among the A^j^, A^r^ and A^n^ subgenomes in *Brassica.* The scales indicate the physical distance for the genome sequences (Mb) of A^r^ and A^n^ subgenomes and the genetic distance for A^j^ subgenome. **a** Inversion events detected among the A^j^, A^r^, and A^n^ subgenomes. The A10 chromosomes from the three subgenomes are shown. Inversion events that occurred in the other chromosomes are given in Additional file 4. The grey lines between the chromosomes indicate consistent alignment, and inversion events are indicated with blue lines. The letters in the body of the chromosomes indicate the large ancestral blocks. **b** Translocation events detected among the A^j^, A^r^, and A^n^. The translocation events involving the A^j^ subgenome are shown with blue lines and the other translocation events are indicated with grey lines inside the circle

The three A subgenomes were compared pairwise by mapping the linkage groups A1 to A10 of *B. juncea* to the genome sequences of the A^r^ and A^n^ subgenomes. As expected, good co-linearity was observed across all the compared chromosomes (Fig. 3a and Additional file 4); however, potential genome-wide chromosomal rearrangement events, both inversions and translocations, were detected among the three subgenomes (Table 2 and Fig. 3a, b). A total of 30 potential inversion events across large segments and 20 potential translocation events were observed for the three comparisons.

**Table 2:** Chromosome rearrangement events detected among three A subgenomes of *Brassica* species

Chromosome	Inversion			Translocation^b^			
	A^j^-A^r^	A^j^-A^n^	A^n^-A^r^	A^j^-A^r^	A^j^-A^n^	A^r^- > A^n^	A^n^- > A^r^
A01	2(2)	2(2)	6(3)^a^	0	0	0	3
A02	3(2)	2(1)	7(6)	0	0	0	4
A03	0(0)	2(0)	4(3)	0	0	2	1
A04	2(0)	1(0)	4(4)	1	1	0	0
A05	1(0)	0(0)	10(7)	0	1	4	3
A06	1(1)	1(1)	3(2)	0	0	1	0
A07	1(0)	1(0)	11(10)	0	0	3	0
A08	1(0)	1(0)	3(3)	0	1	4	2
A09	1(1)	2(1)	8(5)	0	0	1	0
A10	1(0)	3(2)	6(4)	1	0	0	2
Total	13(6)	15(7)	62(47)	2	3	15	15

^a^The numbers indicate the total number of inversion events and the numbers in brackets indicate small inversion events where the segment was below the physical distance of 1 Mb between A^n^ and A^r^, and from 0.6 to 1.2 cM between A^j^ and A^r^/A^n^

^b^The symbol “>” indicates the direction of translocation

### Evolutionary analysis among *Brassica* species with an A subgenome

To further investigate the phylogenetic relationship and population structure between *B. juncea* and the other *Brassica* species with an A subgenome, a set of 47,550 high quality DArT-seq markers was used to genotype 26 accessions selected from *B. juncea*, *B. rapa*, and *B. napus* (Table 3). The level of detected marker polymorphism was 30.6 % and 28.9 %, in the two allotetraploid species *B. napus* and *B. juncea*, respectively, and 19.1 % in the diploid *B. rapa*. The markers were aligned to the published genome sequence of *B. rapa*, *B. napus*, and to the unpublished draft genome sequence of *B. juncea* (Isobel Parkin, personal communication). The 28,267 polymorphic markers were aligned with strict parameters (E-value ≤ 10^−20^ and match length ≥ 60 bp) to the genome sequences, 43 % of the markers aligned to multiple regions, but more than half (16,077) were found to align uniquely to the A (3296), B (8278) and C (4573) genomes, respectively, and were referred to as the ABC-markers.

**Table 3:** The diverse lines of three *Brassica* species carrying A subgenomes analyzed in this study

Code	Species	Accession name	Country of origin	Conservation	Breeding period	Seed quality	Other notes
Bj-1	*B. juncea*	113'68	Germany	Self-pollinated	Before 1980	High EAC^a^, high GLU	Spring type, cultivar
Bj-2	*B. juncea*	Changyanghuangjie	China	DH line	Before 1980	High EAC, high GLU	Spring type, cultivar
Bj-3	*B. juncea*	Huayekucai	China	Self-pollinated	Before 1980	High EAC, high GLU	Spring type, cultivar, Aphid–resistant
Bj-4	*B. juncea*	K-100	Pakistan	Self-pollinated	Before 1980	High EAC, high GLU	Spring type, cultivar
Bj-5	*B. juncea*	SV9041341	Sweden	Self-pollinated	Before 1980	High EAC, high GLU	Spring type, cultivar
Bj-6	*B. juncea*	Silayangka	Russia	Self-pollinated	1980–2000	Low EAC, high GLU	Spring type, cultivar
Yellow-S	*B. juncea*	Sichuan Yellow	China (Southwest)	Self-pollinated	Before 1980	High EAC, high GLU	Spring type, landrace
Purple-M	*B. juncea*	Purple Mustard	China (Central)	Self-pollinated	Before 1980	High EAC, high GLU	Spring type, cultivar
Br-1	*B. rapa*	Chengduaiyoucai	China (West)	Self-pollinated	Before 1980	High EAC, high GLU	Spring type, cultivar
Br-2	*B. rapa*	Xishuiyoucai	China (Central)	Self-pollinated	Before 1980	High EAC, high GLU	Semi-winter type, landrace
Br-3	*B. rapa*	Yangyou2	China (Southeast)	Self-pollinated	Before 1980	High EAC, high GLU	Spring type, cultivar
Br-4	*B. rapa*	B3	China (Central)	DH line	Before 1980	High EAC, high GLU High EAC, high GLU	Winter type, landrace
Br-5	*B. rapa*	Tianmenyoucaibai	China (Central)	Self-pollinated	Before 1980	High EAC, high GLU	Semi-winter type, cultivar
Br-6	*B. rapa*	Chiifu	Korea	Self-pollinated	Before 1980	High EAC, high GLU	Spring type, sequenced cultivar
Br-7	*B. rapa*	Denglongzhong	China	Self-pollinated	Before 1980	High EAC, high GLU	Spring type, landrace
Br-8	*B. rapa*	Qixingjian	China (South)	Self-pollinated	Before 1980	High EAC, high GLU	Spring type, cultivar, low sensentitvie to *Peronospora*
Br-9	*B. rapa*	Shangdangyoucai	China (Northeast)	Self-pollinated	Before 1980	High EAC, high GLU	Winter type, cultivar
Bn-1	*B. napus*	Brauner Schnittkohl	Siberian	Self-pollinated	Before 1980	Unkown	Wild napus, kale
Bn-2	*B. napus*	English Giant	England	Self-pollinated	Before 1980	Unkown	Wild napus, Winter forage rape
Bn-3	*B. napus*	Vige DH1	Sweden	Self-pollinated	Before 1980	Unkown	Wild napus, Winter forage rape
Bn-4	*B. napus*	Sure Regent	Europe	Self-pollinated	1977	High EAC, high GLU	Winter, cultivar
Bn-5	*B. napus*	Bronowski	Poland	Self-pollinated	Before 1970	High EAC, low GLU	Spring type, cultivar
Bn-6	*B. napus*	Bievenun	France	Self-pollinated	1982	Low EAC, high GLU	Winter type, cultivar
Bn-7	*B. napus*	Chuangyou2	China (Southwest)	Self-pollinated	1960–1970	High EAC, high GLU	Semi-winter type, cultivar
Bn-8	*B. napus*	Ningyou1	China (Southeast)	Self-pollinated	1968	Middle GLU, middle EAC	Semi-winter type, cultivar
Bn-9	*B. napus*	Huashang3	China (Central)	DH line	1999	Low EAC, Low GLU	Semi-winter type, cultivar

^a^EAC and GLU represent the abbreviation of seed erucic acid and seed glucosinolate, respectively

**Fig. 4:**
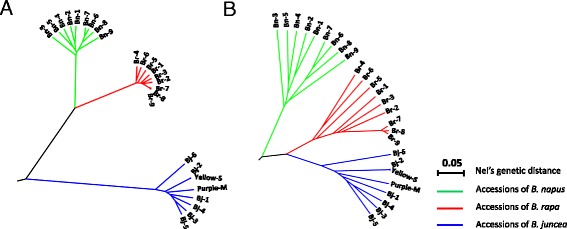
Phylogenetic tree of the three A genome contained species *B. rapa*, *B. juncea* and *B. napus.* **a** Evaluated with a set of markers (16,077) which was aligned to unique positions of A, B and C genomes (ABC-markers), respectively; the figure shown *B. juncea* being separated from other two species. **b** Evaluated with a subset of markers (3296) which were uniquely aligned to A genome; the figure shown a close relationship between *B. juncea* and *B. rapa*, and together separated from *B. napus*. 26 accessions of the three *Brassica* species, i.e., *B. rapa*, *B. juncea* and *B. napus*, were used

Phylogenetic analysis of the three A-genome containing species was studied with the ABC-markers. It was shown that *B. juncea* species was clearly divergent from the other two species, *B. rapa* and *B. napus*, which shared a closer genetic relationship (Fig. 4a). When the subset of A genome specific markers (3296) was used to construct the phylogenetic tree, however, *B. juncea* showed a closer genetic relationship with the A genome species-*B. rapa*, and *B. napus* was separated from its two related A genome-carrying species (Fig. 4b, Additional file 5). Similar results were also found when the two sets of A genome markers (all of the markers with alignment to A genomes including those with multiple alignment to other genomes, and A genome-specific markers) were used to estimate genetic distance for the three species. The A genome in *Brassica* seems differentiated into two clades, A^j^-A^r^ clade and A^n^ clade (Additional file 6).

**Fig. 5:**
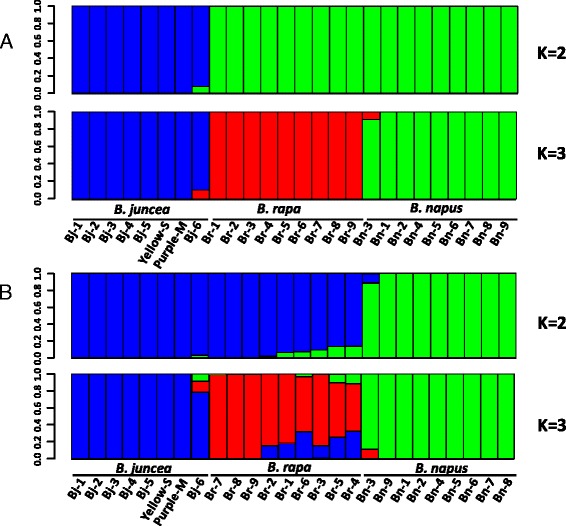
Graphical representation of population genetic structure of the 26 *Brassica* accessions generated using the program STRUCTURE. **a** Population structures were evaluated with a set of markers (16,077) which was aligned to unique positions of A, B and C genomes (ABC-markers). The ∆K values were >3500 and >5000 at K = 2 (up) and K = 3 (lower), respectively (Additional file 7A). **b** Population structures were evaluated with A genome specific markers. The ∆K values were >1000 and ≈ 400 at K = 2 (up) and K = 3 (lower), respectively (Additional file 7B)

Population structure analysis [40] constructed with all the ABC-markers also revealed clear genetic differentiation among the 26 accessions of the three *Brassica* species (Fig. 5a, Additional file 7). At K = 2, *B. rapa* and *B. napus* shared close membership and obviously diverged from *B. juncea*, suggesting a unique gene pool for *B. juncea*, which would be mainly attributed to the B genome composition of *B. juncea*. At K = 3, the three species were separated, however, the *B. juncea* cultivar Silayangka (code No. Bj-6) from Russia and a breeding accession Vige DH1 (code No. Bn-3) of *B. napu*s from Sweden shared part membership with *B. rapa*, suggesting gene flow or introgression from subgenome A^r^ to A^j^ and A^r^ to A^n^ may have occurred. When we evaluated the genetic structure using the A genome specific markers, the highest ∆K value was observed at K = 2 (∆K > 1000), and the two clades, A^j^-A^r^ clade and A^n^ clade, appeared again as in the phylogenetic analysis (Fig. 5b). However, gene flow between the A^j^-A^r^ clade and the A^n^ clade was also suggested, in particular from A^n^ to A^r^. Gene flow among different A subgenomes was more obvious at K =3 (^∆^K ≈ =400) where a strong gene flow of A^j^ > A^r^ was suggested, while the *B. juncea* cultivar Silayangka, contrarily, seems to have received introgressions from two subgenomes, A^r^ and A^n^ (Fig. 5b, Additional file 7).

## Discussion

High-density accurate genetic maps are essential for quantitative trait locus (QTL) analyses, ordering of sequences on physical maps (genome assembly), map-based cloning of genes, and comparative mapping across populations within and between species. Population size, missing data, and segregation distortion of markers can affect the accuracy of a genetic linkage map [41]. Although the original set of polymorphic markers detected with relatively high quality was as high as 6836 (with missing rate ≤ 10 % and reproducibility scores >90 %), only 70 % of the markers (4833) with high confidence (with missing data rate ≤ 5 % or could be classified in genetic bins represented by markers with less than 5 % missing rate) were selected to construct the linkage genetic map of *B. juncea*, to ensure the quality of the genetic map. To retain the sequence information associated with as many of the 1570 mapped loci as possible, the concept of “bin loci” was used [42] allowing a total of 3329 markers to be anchored on the map (Additional file 2).

Of the 24 ancient karyotype blocks, 23 were detected in the SY-PM genetic map. The G block was not found perhaps because of the limited genetic diversity on the G blocks between the parents of the SY-PM mapping population and/or the short physical length of the G blocks in the *A. thaliana*, *B. rapa*, and *B. napus* genomes. We calculated that, compared with the other ancestral blocks, the G block was small in the *A. thaliana* (1.6 Mb) and much smaller in *B. napus* (0.8 Mb) genomes. Only a very few markers identified among the 26 accessions of the three species could be aligned to the ancestral block G of Brassicaceae, and also only a few G blocks have been identified in *Brassica* genomes previously [2, 6–9, 13, 15, 16, 29, 43] (Additional file 3). These findings suggested that the genetic diversity of the G blocks might be limited in *Brassica* species, or a feature of the G block is evolutionarily conserved, which leads to few informative markers available for comparative analysis.

It is interesting and important to understand the evolutionary processes and genomic variation that occurred in the A, B, and C genome of *Brassica* species that were domesticated by our Neolithic ancestors. Since the sequence information of A^j^B^j^, B^ni^ and B^c^C^c^ genomes is currently not yet available in public domain, we concentrated on genetic comparisons among A genome containing *Brassica* species. In this study, the comparative analysis based on the macro-constitution of the 24 conserved ancestral blocks suggested that these ancestral blocks may have undergone different evolutionary processes which apparently lost or increased certain blocks in the three A genome-carrying *Brassica* species relative to the common ancestor (Table 2, Additional file 3). From the three pair-wise comparisons between the three A genome-carrying species, assisted with a comparative genetic map and genome sequence analysis, we found considerable inversion and translocation events among the three A subgenomes (Table 2, Additional file 4). Greater genomic differentiation and more inversion and translocation-like events were observed here in comparisons between A^r^ and A^n^ than that between A^j^ and A^r^, or between A^j^ and A^n^. It is to note that the resolution is greater when estimating divergence between A^r^ and A^n^ based on their genome sequence assemblies than with the A^j^ genome based on the genetic map. However, we may have over-estimated or under-estimated some of the structural variation, since it could also result from mistakes during the genome sequence assembly. Despite this, the observed variation of ancestral blocks and chromosome structure among the three A genomes suggests that the three A subgenomes are differentiated by considerable genomic variation even though they share strong co-linearity. These data provide insights into the evolutionary processes that differentiate the diploid *Brassica* sub-genomes, which will be more comprehensively analyzed and understood when the genome sequence of *B. juncea*, *B. nigra*, *B. carinata* will be available.

Increasing evidence has shown that the B genome diverged from the A/C genome about 6–7 million years ago (Mya), while the A genome of *B. rapa* and C genome of *B. oleracea* may have diverged about 3.7 Mya, as determined by co-linearity and *Ks* analyses across the genomes [6, 16, 21, 44–46]. The population genetic analysis of 26 accessions of the three A genome-carrying *Brassica* species with a common set of ABC-markers showed three clear sub-populations representing the three species, indicating *B. juncea* was divergent from *B. rapa* and *B. napus*, which is likely mainly attributed to the B genome composition in *B. juncea* and the C genome composition in *B. napus* (Fig. 4a, Additional file 5, Fig. 5a). These results also supported previous hypothesis that the B genome diverged earlier from the A/C genome into an independent lineage. Interestingly, when the accessions of the three species were evaluated with A-genome-specific markers, two major clades, A^j^-A^r^ clade and A^n^ clade, were observed, which was quite different from the result evaluated using the ABC-markers (Figs. 4b and  5b, Additional file 5). These results were consistent with the hypothesis that both species of *B. rapa* and *B. juncea* originated from a close geographical region or the same center of origin, Asia, and have closer A subgenomes, whereas *B. napus* originated from Europe as a result of spontaneous hybridization between *B. rapa* and *B. oleracea* [6, 9, 16, 23, 34]. More accessions of *B. rapa* originating from Europe should be examined in the future. Herein, we used old cultivars and landraces of Asian origin (Table 3), which might have been less frequently involved in gene introgression. Additionally, the gene flow observed between the A^j^-A^r^ clade and the A^n^ clade as shown in Fig. 5 suggested the introgression of A^r^ to A^j^ or A^n^ during the breeding process of the development of cultivars.

Subgenomic differentiation in *Brassica* may be significant in creating novel types in *Brassica* species that could help broaden the genetic diversity or increase heterosis. Considerable effort has focused on exploring introgression from the A genome of *B. rapa* or *B. napus*, C genome of *B. oleracea* or *B. carinata* into *B. napus* or *B. juncea* [26, 47–52]. Our results support the idea that *B. juncea* has distinct genomic diversity, and/or evolved from a different A genome progenitor than *B. napus*. We suggest that variation in the A^j^ subgenome of *B. juncea* could be applied to the genetic improvement of *B. napus*. Additionally, the possible divergence between the A genome progenitor of *B. napus* and Asian *B. rapa* accessions would suggest that these lines would be very useful to broaden the genomic diversity of *B. napus*.

The dense genetic linkage map and the comparative analysis of three A subgenomes described here, will contribute to a better understanding of *Brassica* subgenomics. This will also help in a comprehensive understanding of variation in the A, B and C genomes and efforts towards genome sequencing of *B. juncea*, *B. carinata* and *B. nigra*.

## Conclusions

Here, we described the construction of a dense genetic linkage map of *B. juncea* based on a DArT-seq approach and compared the genome constitution of the 24 ancestral blocks of Brassicaceae and the chromosome rearrangement events among three A-genome carrying *Brassica* species (*B. rapa*, *B. juncea*, and *B. napus*). The genetic map of *B. juncea* was constructed using an F_2_ population derived from two Chinese cultivars SY and PM, and contained 3329 DArT-seq markers at 1570 genetic loci. We identified 18 linkage groups that covered a genetic distance of 1579 cM and detected 23 of the 24 conserved ancestral blocks of Brassicaceae in the SY-PM genetic map. The chromosome rearrangements among the three A subgenomes revealed by genetic mapping and genome sequence comparative analysis, and the population genetic analysis of diverse lines of the three A-genome carrying *Brassica* species, showed that the A^j^ subgenome of *B. juncea* had a close relationship with A^r^ subgenome, and was quite different from the A^n^ subgenome. Our results support the idea that *B. juncea* has distinct genomic diversity, and/or evolved from a different A genome progenitor than *B. napus*, which could be exploited for genetic improvement of *B. napus*. The dense genetic map presented here will help in generating reference sequences of the different U’ triangle [25] genomes and subgenomes and would further facilitate the (i) elucidation of genomic differentiation as a result of speciation, evolution, and adaptation of different *Brassica* species, and (ii) the transfer of favorable alleles between species to develop improved varieties to meet the global demand for vegetable oils.

## Methods

### Plant materials and development of the genetic mapping population

Two Chinese landraces of the oilseed *B. juncea*, Sichuan Yellow (abbreviated as ‘SY’) and Purple Mustard (abbreviated as ‘PM’), were used to construct an F_2_ genetic mapping population (abbreviated as ‘SY-PM’). Both parental lines were subjected to self-pollination for eight successive generations. The maternal parent, ‘SY’, is a local variety with yellow seed color from Sichuan Province, the southwest of China. The paternal parent, ‘PM’ is a local variety with black seed color from Hunan Province, in the central region of China [37]. One individual ‘SY’ plant was pollinated by ‘PM’. A single F_1_ plant from this cross was self-pollinated, and 168 F_2_ plants were obtained. This F_2_ population (SY-PM) was used for genetic linkage map construction.

A total of 26 relatively old accessions representing the A genomes of the major cultivated oilseed *Brassica* species, mostly Asian *B. juncea* and *B. rapa,* and *B. napus* collected from different countries, were used in the comparative analysis (Table 3).

### Genotyping

Whole-genome profiling of the SY-PM population and 26 accessions of the three A-genome carrying *Brassica* species was performed using the DArT-seq at Diversity Arrays Technology Pty. Ltd. (DArT P/L, Canberra, Australia). DNA extraction and genotyping by DArT-seq technique were performed as described previously [15, 39]. We used genomic DNA (50 ng/μl) isolated from fresh and young leaves. DArT-seq was performed using the HiSeq2000, next-generation sequencing platform (Illumina, USA). Both SNPs and presence-absence polymorphisms, collectively called ‘DArT-seq markers’, were identified by DArT P/L. The 69-bp long DArT-seq markers sequences were used for the comparative genome analysis. We identified a total of 10,174, and 81,372 sequence variants comprising both *in silico* DArTs (referred to as present/absent markers) and SNP markers in the SY-PM genetic mapping population, and the 26 accessions of the three species, respectively. We used markers that had overall call rates >90 % for present/absent markers (percentage of valid scores of all possible scores for a marker), call rates >90 % and Q values >2.0 for SNP markers (the logarithm of the minimum false discovery rate at which the test may be called significant), and percentages of the missing data or errors in the diverse population of ≤5 % and mapping population of ≤10 %. Finally, a total of 6836 and 47,550 high quality DArT-seq markers were filtered out in the SY-PM population and 26 diverse lines, respectively.

### Genetic map construction and genomic comparative analysis

To construct the genetic map, we first used 6836 polymorphic markers (with call rate (>90 %) and missing rate ≤10 %) identified in the SY-PM mapping population to classify genetic bins. We discarded those genetic bins totally consisting of the markers with more than 5 % missing rate. Therefore, 4833 markers were remained for constructing genetic map. Secondly, we choose representative markers with the least missing data from each “bin locus” together with the single markers (1132) with less than 5 % missing rate to construct the genetic map. The genetic map of the SY-PM population of *B. juncea*, was constructed using JoinMap (version 4.0) [53]. The genetic mapping process and the parameters were described previously [15]. Distorted segregation of markers was analyzed using the *χ*^2^ (Chi-square) test according to the expected segregation ratio for presence/absence of markers and SNP variants in an F_2_ population. To estimate the impact of distortion on the accuracy of the marker order on the genetic map, we compared the genetic position of each locus in the SY-PM genetic map with and without the inclusion of the distorted markers. Almost the same order of loci, but with genetic distances that changed by under 0.6 cM, was observed in the different versions of the genetic maps when the majority of distorted markers was added or deleted for map construction. Occasionally, inversions covering 0.1-1 cM of a genomic region were detected when distorted markers were added. These markers were deleted to construct an accurate genetic map. We used a pragmatic approach to include distorted markers that did not alter the order of markers within linkage groups. Unlikely “double recombinants” were also checked and discarded for genetic mapping. Linkage groups were assigned according to their alignment with the *B. rapa* and *B. napus* genome sequences [23, 38] and the positions of the DArT-seq markers on the *B. carinata* genetic map [15]. Since the recombination events were limited with the population, some of the markers could be mapped on the same genetic position at the linkage map because of no-recombination, and therefore, we defined such markers as “bin markers” and such genetic position as “a bin locus” [42]. All of the high quality markers recorded in the SY-PM population and 26 diverse lines with known sequence were used in BLAST searches (with default parameters) against the *A. thaliana* (TAIR9 genome release, ftp://ftp.arabidopsis.org/home/tair/Genes/TAIR9_genome_release/TAIR9_chr_all.fas)*, B. rapa* [38], and *B. napus* [23] genome sequences, and the unpublished genome sequence of *B. juncea* provided by Isobel AP Parkin (Agriculture and Agri-Food Canada, Canada).

We set the E-value ≤ 10^−6^ and the match length to over 40 bp (DArT-seq marker sequence was 69 bp in length), to filter the matches to ancestral blocks for SY-PM genetic mapping population. A total of 659 marker sequences in the SY-PM genetic map could be aligned to the ancestral block. Meanwhile, by aligning the marker sequence to the “Darmor” genome of *B. napus* [23], an additional 156 marker sequence could be assigned to certain ancestral blocks. The marker sequence were finally assigned to certain ancestral blocks according to the best match length, E value, good alignment consistency with adjacent loci, good alignment consistency among bin markers, good alignment consistency with other published genetic map of *B. juncea*. As a result, 815 marker sequences representing 767 loci on the SY-PM genetic map were aligned with the sequences of *A. thaliana* for further analysis. At least three consecutive homologous loci assigned to the same ancestral block were defined as a synteny block [15], and only one or two closest loci assigned to the same ancestral block with nearly 100 % identity were considered as a small segment of insertion. Using homology searches against the *Arabidopsis* pseudo-chromosomes as described previously [15], the 24 ancestral blocks (A to X) that are conserved in Brassicaceae family and be organized to generate the eight chromosomes of the ancestral crucifer karyotype [19], were identified in the genetic map of the SY-PM population. The chromosomal constitution of the A subgenome of *B. juncea* was compared with the two A subgenomes of *B. rapa* and *B. napus* as described previously [2, 29].

The DArT-seq marker sequence were only 69 bp in length and therefore likely to generate a large number of alignments for the SY-PM genetic mapping population and the 26 accessions of the three species in which A, B and C genomes contained. To identify the most appropriate alignment to the *Brassica* genome sequence for each maker sequence, we set the E-value ≤ 10^−20^ and the match length ≥ 60 bp to filter the match. We therefore used the match for further genome comparison analysis between three A subgenomes with the SY-PM genetic map. We also discerned those markers uniquely aligned to A, B and C genomes, and those markers with multiple alignments to different genomes, respectively, which were used for the population genetic analysis for the diverse lines of the three A genome-carrying species.

Genomic changes such as inversion and translocation events among the three A genome pairs, A^j^-A^n^, A^j^-A^r^, and A^r^-A^n^, were identified by comparative genetic mapping using the loci with known sequence information in the linkage groups of the A genome of the SY-PM population and their corresponding alignment matches in the A^r^ and A^n^ genomes. Based on the *B. juncea* (SY-PM) population size in this study (168 lines), a genetic distance of 0.6 cM (100/168), would be the shortest permitted genetic distance within its resolving power according to the criterion of one ‘cM’. Considering the possibility of genetic mapping errors resulting from missing data and double crossing-overs, a valid inversion event was recognized if the order of the marker cluster was reversed over a genetic distance that was greater than the permitted distance for error (i.e., 0.6 cM in the SY-PM genetic map). At least two loci should be inverted in a valid inversion, and we did not discern the too complicated inverted loci without consistent inverted order. At the same time, the alignment information of the markers included in the same genetic bins was also used to judge the order of the loci. An inversion that occurred between the genetic distance (0.6 cM) and double genetic distance (1.2 cM), was identified as an inversion event in a small segment, and an inversion that covered more than 1.2 cM was identified as an inversion event in a large segment. A genetic distance of 0.6 cM in the linkage groups of the A genome would correspond to a physical distance of about 1 Mb, based on the size of the A genome (approximately 485 Mb) and the total map length of the A^j^ linkage groups on the SY-PM genetic map. Therefore, the physical distance of 1 Mb was used as the critical distance to discern an inversion event happened within small segments or within large segments of the genome. For discerning translocation event between A^r^ to A^n^ by comparison with genome sequence, a valid translocation event was discerned if the segment with more than four successive adjacent genes was aligned to different chromosome of the other A subgenomes. If we observed markers located in one locus or adjacent loci of A^j^, but they were mapped in different chromosomes of A^r^ or A^n^ with 100 % unique identity alignment to these chromosomes, we counted a candidate translocation event between A^j^ and A^r^/A^n^. To confirm the stability of marker order around the detected translocations, we randomly moved the neighbor markers to reconstruct genetic maps and compare the order of the common markers between the maps (before and after moving neighboring markers), the potential translocation events were then finally ‘declared’ if there was no change of the order of the common markers.

### Genetic diversity and population structure analysis

A total of 47,550 high quality DArT-seq variants, were scored in 26 accessions of the three A genome-carrying species (*B. rapa*, *B. juncea* and *B. napus*) and used for further analysis. Genetic dissimilarities among diverse genotypes were estimated using Nei’s coefficient [54] and used for the phylogeny reconstruction using the UPGMA (unweighted pair-group method with arithmetic means) method implemented in PowerMarker version 3.25 [55]. The phylogenetic tree was viewed using MEGA 4.0 [56]. Analysis of the population structure among the accessions was performed using STRUCTURE version 2.3.4 [40]. The length of burn-in time and replication number were both set to 100,000 in each run. To identify and determine the most probable number of populations (K), we calculated the ^∆^K values of K from 1 to 10 replicate runs for each K. Finally, we selected the K value that corresponded to the peak of the ^∆^K graph after plotting [57].

### Availability of supporting data

All the supporting data are included as additional files. Phylogenetic data is available in the TreeBASE as accession number S18584 (http://purl.org/phylo/treebase/phylows/study/TB2:S18584), and more detailed information for the marker sequence alignment and phylogenetic analysis could be also available from the Dryad Digital Repository: http://dx.doi.org/10.5061/dryad.56986.

## Additional files

Additional file 1:
**Information of DArT-seq markers and genotypes of the SY-PM genetic mapping population of** ***B. juncea.*** (XLSX 6076 kb) Additional file 2:
**Information of the linkage map of the** ***B. juncea*** **constructed with SY-PM population.** (XLSX 1052 kb) Additional file 3:
**The copies of ancestral blocks of Brassicaceae discriminated in** ***Brassica*** **species and** ***Brassica*** **subgenomes.** (XLSX 15 kb) Additional file 4:
**Comparison between the three A subgenomes.** Ten sheets present the comparison from A1 to A10 among the three A subgenomes. The lines with blue color in one segment show an inversion event. (XLSX 2894 kb) Additional file 5:
**Phylogenetic tree of the three A genome-contained species evaluated with different sets of markers.** A, Evaluated with all of high-quality markers with genome alignments (28,267) (left), with a set of markers (16,077) which was aligned to unique positions of A, B and C genomes (ABC-markers, middle), and markers (12,781) which were uniquely aligned to B (8278) and C (4503) genomes(right), respectively. All of the three figures shown that *B. juncea* was separated from other two species. B, Evaluated with markers aligned to A genome uniquely (3296) (left), and 3296 A genome specific markers plus 11,321 markers with alignment on A genome along with positions on other genome(s). *B. juncea* and *B. rapa* shown close relationship and separated from *B. napus*. (PDF 552 kb) Additional file 6:
**Calculation on the population genetic distance among the three species carrying A genome by resampling.** (XLSX 30 kb) Additional file 7:
**The ∆K value of the population genetic structure of the 26** ***Brassica*** **accessions generated using the STRUCTURE program calculation with different data set of markers.** A, Evaluated with a set of markers (16,077) which was aligned to unique positions of A, B and C genomes (ABC-markers). The highest ∆K value was observed at K = 3, where K is the most probable number of populations. An inflexion high ∆K value was also observed at K = 2, which also indicated the genetic relationship among the diverse lines. B, Evaluated with markers aligned to A genome uniquely (3296). The highest ∆K value was observed at K = 2, and an inflexion high ∆K value was also observed at K =3, which also indicated the genetic relationship among the diverse lines. (PDF 162 kb)

## Abbreviations

AFLP: amplified fragment length polymorphism
*B. juncea*: *Brassica juncea*
bp: basepair
cM: centiMorgan
DArT P/L: Diversity Arrays Technology Pty. Ltd.
EAC: seed erucic acid
GLU: glucosinolate
LG: linkage group
Mb: megabase
MYA: million years ago
PM: purple mustard
QTL: quantitative trait locus
RFLP: restriction fragment length polymorphism
SNP: single nucleotide polymorphism
SSR: simple sequence repeats
SY: sichuan yellow
UPGMA: unweighted pair-group method with arithmetic means
*χ*^2^: Chi-square
