# Supplementary figures and images for: Co-linearity and divergence of the A subgenome of Brassica juncea compared with other Brassica species carrying different A subgenomes
Source: BMC Genomics. 2016 Jan 5;17:18. doi: 10.1186/s12864-015-2343-1 (PMC4700566; doi:10.1186/s12864-015-2343-1)

A

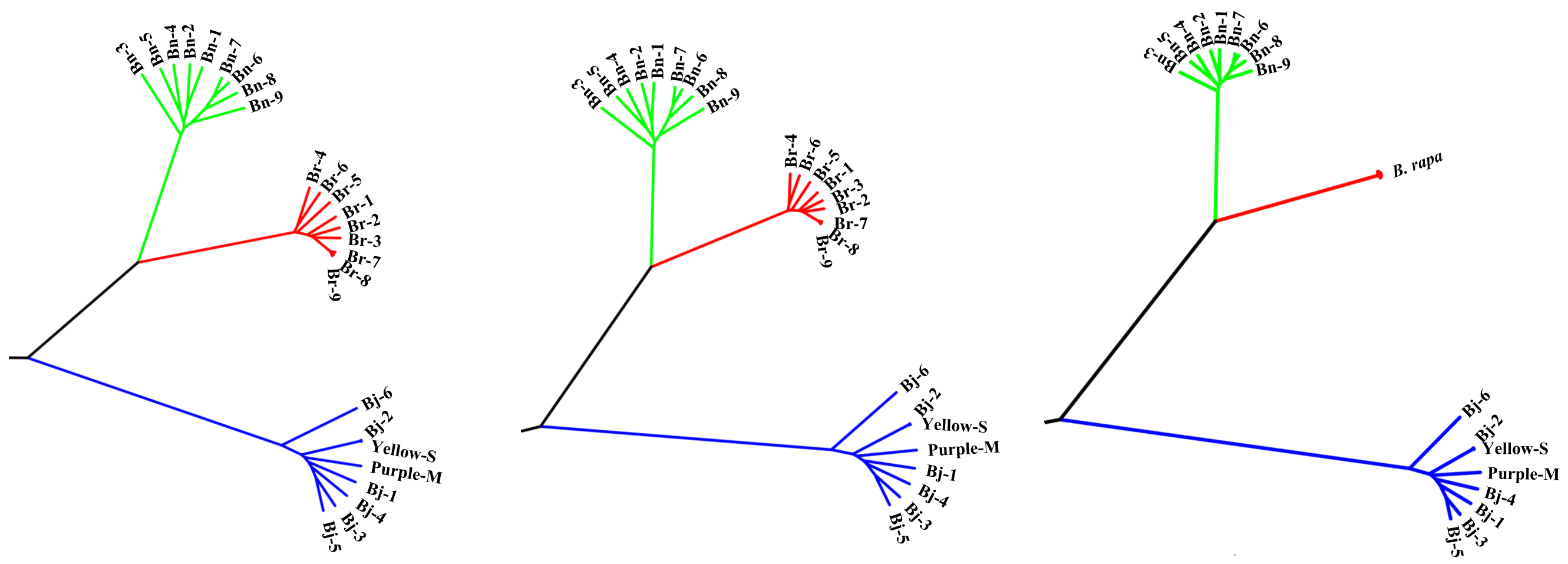

B

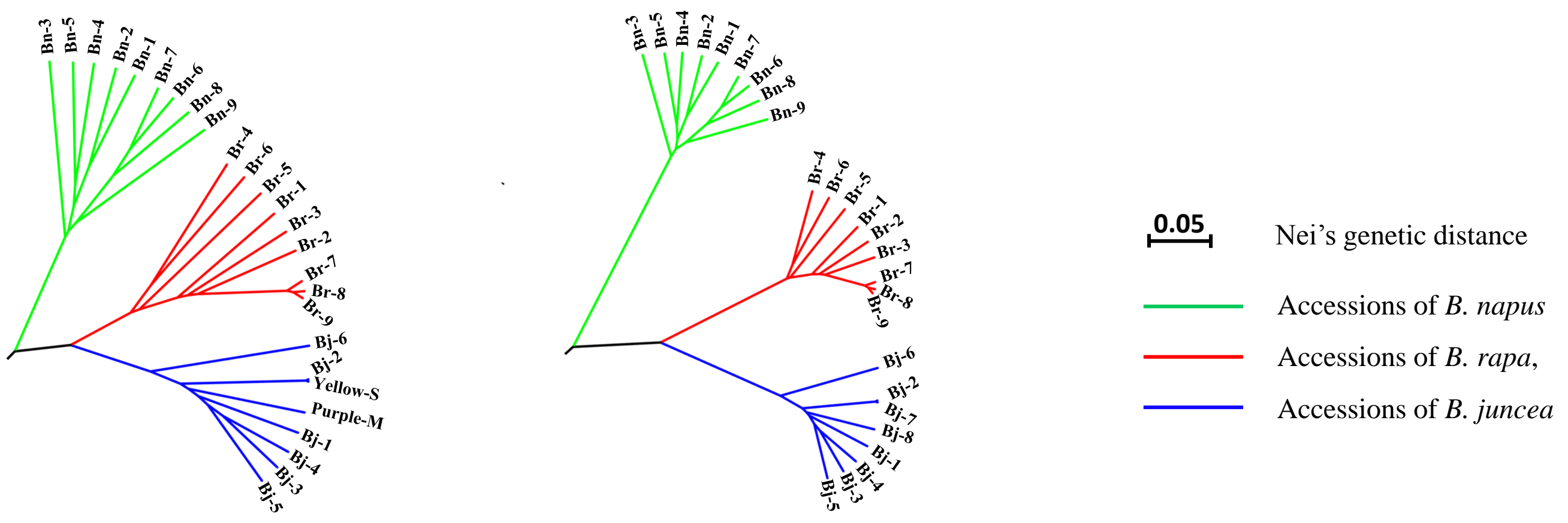

Supplement: Additional file 5: — Phylogenetic tree of the three A genome-contained species evaluated with different sets of markers. A, Evaluated with all of high-quality markers with genome alignments (28,267) (left), with a set of markers (16,077) which was aligned to unique positions of A, B and C genomes (ABC-markers, middle), and markers (12,781) which were uniquely aligned to B (8278) and C (4503) genomes(right), respectively. All of the three figures shown that B. juncea was separated from other two species. B, Evaluated with markers aligned to A genome uniquely (3296) (left), and 3296 A genome specific markers plus 11,321 markers with alignment on A genome along with positions on other genome(s). B. juncea and B. rapa shown close relationship and separated from B. napus. (PDF 552 kb) [file 12864_2015_2343_MOESM5_ESM.pdf]

A

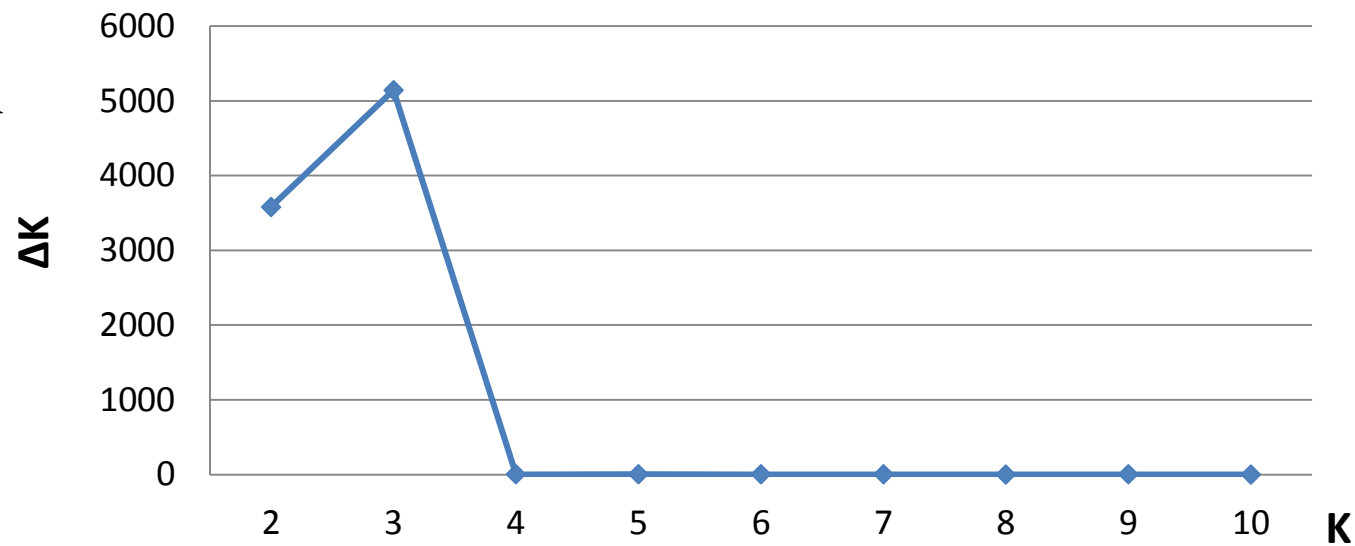

B

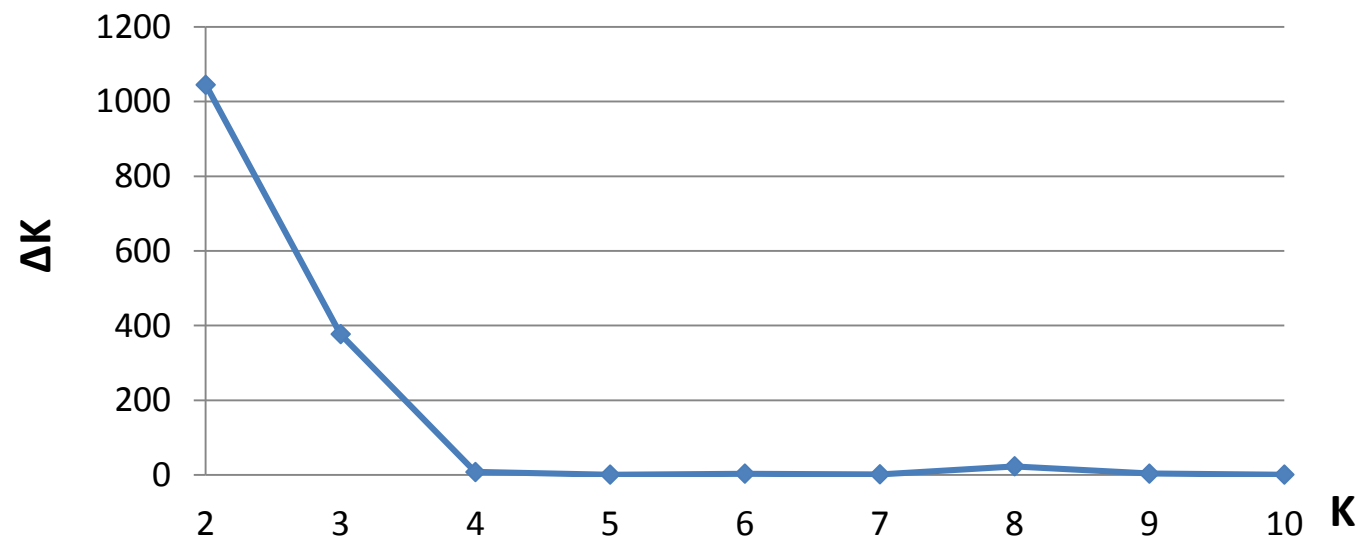

Supplement: Additional file 7: — The ∆K value of the population genetic structure of the 26 Brassica accessions generated using the STRUCTURE program calculation with different data set of markers. A, Evaluated with a set of markers (16,077) which was aligned to unique positions of A, B and C genomes (ABC-markers). The highest ∆K value was observed at K = 3, where K is the most probable number of populations. An inflexion high ∆K value was also observed at K = 2, which also indicated the genetic relationship among the diverse lines. B, Evaluated with markers aligned to A genome uniquely (3296). The highest ∆K value was observed at K = 2, and an inflexion high ∆K value was also observed at K =3, which also indicated the genetic relationship among the diverse lines. (PDF 162 kb) [file 12864_2015_2343_MOESM7_ESM.pdf]
